# Editorial: Microsurgical Anatomy of the Central Nervous System and Skull Base

**DOI:** 10.3389/fsurg.2021.794679

**Published:** 2021-11-18

**Authors:** Gustavo R. Isolan, Alexander I. Evins, Ricardo Lopes De Araujo, Antonio Bernardo

**Affiliations:** ^1^Centro Avançado de Neurologia e Neurocirurgia (CEANNE), Porto Alegre, Brazil; ^2^Faculdade Evangelica Mackenzie Do Parana, Curitiba, Brazil; ^3^Weill Cornell Medicine, Neurological Surgery, New York, NY, United States

**Keywords:** surgical anatomy, neurosurgery, central nervous system, microneurosurgery, neurosurgical approaches

Since the times of Da Vinci and Vesalius, dissections of the human nervous system have played a fundamental role in uncovering its multitude of functions. In the ensuing centuries, neuroanatomical dissections have evolved from studying the basic structure and nature of the nervous system to advancing our current understandings of neuropathology, neurophysiology, and neurosurgery (1–3). Today, neuroanatomical dissections remain the best avenue for complex skills and knowledge development in neurosurgery and are essential for expanding the neurosurgical armamentarium.

A thorough topographical understanding of anatomical structures and their three-dimensional spatial relationships is essential in order to develop the skills required to maximize surgical corridors to deep intracranial targets (1). As most surgeons limit their anatomical knowledge to well established surgical corridors, anatomically oriented neurosurgical training unfortunately remains lacking and inadequate in training programs worldwide (1). The complex nature of neurosurgical anatomy necessitates enhanced preoperative anatomic training, and it is our firm belief that microanatomical proficiency translates into surgical proficiency, which in turn directly influences patient outcomes. This is especially true when performing surgery of the skull base. To that point, we believe that for surgical education and research, the human specimen is the ultimate resource and cannot be replaced by any current technology or textbook.

As such, we are pleased to present this special Research Topic on Microsurgical Anatomy of the Central Nervous System and Skull Base. The works included in this topic not only highlight the importance of anatomical dissection in neurosurgery but demonstrate the value of such research to improving patient care and outcomes.

The first article in this topic by Chen et al. examines the microsurgical knowledge and strategies used in the management of anterior communicating artery aneurysms. The authors review various studies from recent literature and find that microsurgical clipping may be the ideal treatment for a large group of patients who present technical challenges to embolization. The authors explore the relationships of the anterior communicating arteries with adjacent anatomical structures and present an up-to-date review of surgical techniques and prognostic factors in the management of anterior communicating artery aneurysms.

The second article by Constanzo et al. provides a detailed review of the microsurgical anatomy of the jugular foramen, as well as the strategies necessary for successful jugular glomus tumor surgery. This is one of the most complex areas of skull base surgery, and the article focuses on the microsurgical anatomy of the craniocervical approach which is necessary for the treatment of these tumors. The article includes highly illustrative intraoperative photos, anatomical dissections, and illustrations.

The next article is an original study by Li et al. wherein the authors present a framework for ultrahigh-resolution digitized mapping of the normal rat spinal cord angioarchitecture and determine physiological parameters using synchrotron radiation micro-CT. This new platform could be an important tool for pre-clinical *ex-vivo* investigations of neurovascular networks.

Finally, focusing on the functional anatomy of the eloquent areas of the central nervous system, Soldozy et al. present an important systematic review of the use of brain mapping to assess functional reorganization and its impact on patients with arteriovenous malformation. The authors conducted a detailed analysis of 12 selected studies and found that cortical reorganization is a common phenomenon in patients with arteriovenous malformations. The authors demonstrate that preoperative mapping impacts AVM grading and, therefore, operative decision making.

We hope that the readership will find these articles and this Research Topic to be a useful reference for emerging research anatomically based research in the neurosciences.

## Author Contributions

GI, AE, and RL contributed to conception and design of the manuscript. GI and AE wrote the first draft of the manuscript. GI, AE, RL, and AB wrote sections of the manuscript. All authors contributed to manuscript revision, read, and approved the submitted version.

## Conflict of Interest

The authors declare that the research was conducted in the absence of any commercial or financial relationships that could be construed as a potential conflict of interest.

## Publisher's Note

All claims expressed in this article are solely those of the authors and do not necessarily represent those of their affiliated organizations, or those of the publisher, the editors and the reviewers. Any product that may be evaluated in this article, or claim that may be made by its manufacturer, is not guaranteed or endorsed by the publisher.
